# The role of circular RNAs in regulating cytokine signaling in cancer

**DOI:** 10.1002/2211-5463.70051

**Published:** 2025-05-12

**Authors:** Vandana Joshi, Swati, Amit Mishra, Amaresh Panda, Vivek Sharma

**Affiliations:** ^1^ Department of Biological Sciences Birla Institute of Technology and Science Pilani, Hyderabad Campus India; ^2^ Cellular and Molecular Neurobiology Unit Indian Institute of Technology Jodhpur India; ^3^ Institute of Life Science (ILS) Bhubaneswar India

**Keywords:** cancer, chemokines, circular RNAs, cytokines, TGF‐β, VEGF

## Abstract

Dysregulation of cytokine signaling is central to the development and progression of cancer. Cytokines are not only involved in promoting cancer development but also regulate anti‐tumor immune responses. Circular RNAs (circRNAs) are single‐stranded, covalently closed RNA molecules lacking free ends, which have emerged as critical regulators of cytokine signaling. Transcriptional and post‐transcriptional regulation of cytokine signaling by circRNAs contributes to cancer pathogenesis. Here, we discuss the emerging role of circRNAs in modulating cytokine signaling pathways that regulate cancer development. In particular, we examine the role of circRNAs in TGF‐β, IL‐6, IL‐10, TNF‐α, VEGF, FGF, PDGF, and chemokine signaling in cancer.

## Graphical abstract

Cytokines present in the tumor microenvironment fuel cancer development. Aberrant expression of circRNAs contributes to cancer progression. Cytokines are involved in regulating circRNA biogenesis. Furthermore, aberrantly expressed circRNAs regulate the expression of ligands, receptors, and downstream effectors involved in cytokine signaling to promote hallmarks of cancer such as inflammation, EMT, proliferation, angiogenesis, and drug resistance.

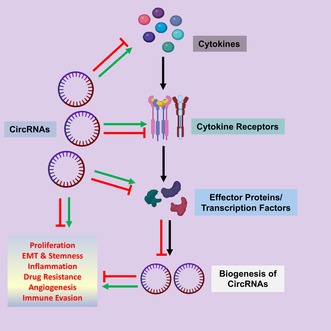

**Abbreviations**
BC: breast cancer
BLCA: bladder cancer
CAF: cancer‐associated fibroblast
CC: cervical cancer
ceRNA: competitive endogenous RNA
CircRNA: circular RNA
CRC: colorectal carcinoma
CTL: cytotoxic T‐lymphocyte
EC: endometrial cancer
ECM: extracellular matrix
EMT: epithelial‐to‐mesenchymal transition
ESCC: esophageal squamous cell carcinoma
FAP: fibroblast activation protein
FGF: fibroblast growth factor
GBM: glioblastoma
GC: gastric cancer
GSC: glioma stem cells
HCC: hepatocellular carcinoma
ICC: intrahepatic cholangiocarcinoma
IFN: interferon
IRES: internal ribosome entry site
LUAD: lung adenocarcinoma
MMP: matrix metalloprotease
NSCLC: non‐small cell lung cancer
OC: ovarian cancer
ORFs: open reading frames
OSCC: oral squamous cell carcinoma
PC: prostate cancer
PDAC: pancreatic ductal adenocarcinoma
PDGF: platelet‐derived growth factor
RBP: RNA‐binding protein
SCLC: small‐cell lung cancer
TAM: tumor‐associated macrophage
TGF‐β: transforming growth factor‐beta
TME: tumor microenvironment
TNBC: triple‐negative breast cancer
TNF‐α: tumor necrosis factor‐alpha
VEGF: vascular endothelial growth factor

## Cytokines

**Fig. 1:**
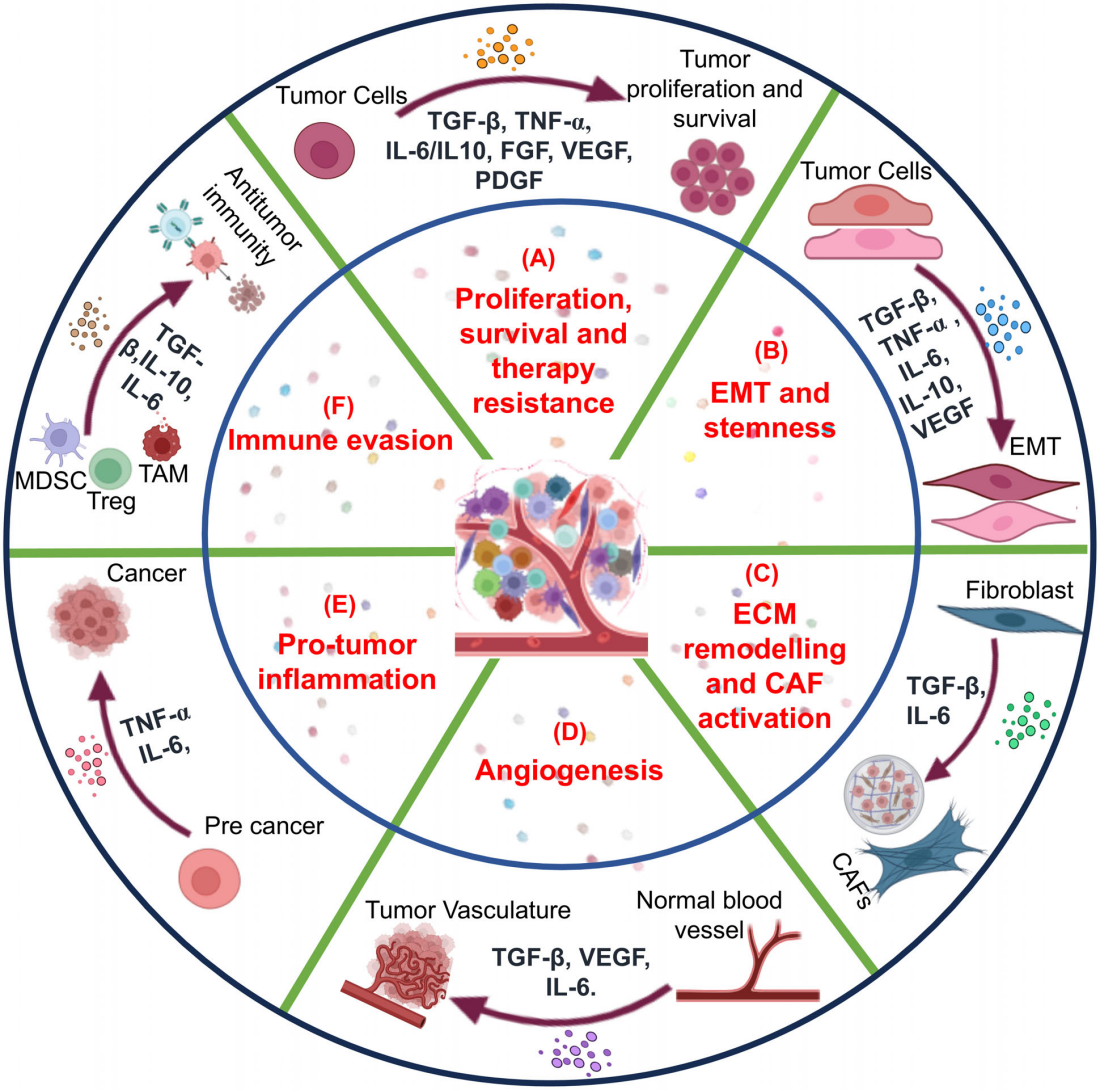
The role of cytokines regulating cancer pathogenesis: (A) TGF‐β, TNF‐α, IL‐6, IL‐10, VEGF, FGF, and PDGF (produced by cancer and other cells of the TME) promote proliferation, survival, and therapy resistance. (B) TGF‐β, TNF‐α, IL‐6, IL‐10, and VEGF promote EMT and stemness. (C) TGF‐β and IL‐6 promote ECM remodeling and fibroblast activation. (D) TGF‐β, IL‐6, and VEGF stimulate the formation of new blood vessels. (E) TNF‐α and IL‐6 promote pro‐tumor inflammation. (F) Anti‐inflammatory activities of cytokines like IL‐10, IL‐6, and TGF‐β regulate immune evasion.

Cancer progression is fueled by its microenvironment composed of stromal cells, endothelial cells, cancer‐associated fibroblasts (CAFs), tumor‐associated macrophages (TAMs), tissue‐resident immune cells, and the extracellular matrix (ECM) [1, 2]. Cytokines are relatively small molecular weight soluble proteins that regulate immune responses and cellular behavior; they are produced by cancer cells and other cells in the tumor microenvironment (TME) (Fig. 1) [3, 4, 5, 6, 7, 8, 9, 10, 11, 12]. TME often shows aberrant cytokine expression and signaling, and it modulates the recruitment of immunosuppressive cells (MDSCs, T‐regs, and TAMs) in the TME to regulate immune evasion and tumor growth [1, 2, 3, 4, 5, 6, 7, 8, 10, 11, 12]. Abnormal paracrine or autocrine signaling by cytokines such as transforming growth factor‐beta (TGF‐β), interleukins, tumor necrosis factor‐alpha (TNF‐α), vascular endothelial growth factor (VEGF), fibroblast growth factor (FGF), platelet‐derived growth factor (PDGF), and interferons (IFNs) modulates cell proliferation, epithelial‐mesenchymal transition (EMT), angiogenesis, invasion, metastasis, cancer stemness, immune response, and response to therapy (Fig. 1) [3, 4, 5, 6, 7, 8, 9, 10, 11, 12]. Chemokines (or chemotactic cytokines) are the largest subfamily of cytokines, which not only regulate chemotaxis to control the infiltration of immune cells in tumors but also directly target tumor and stromal cells to promote cancer progression [3, 4, 5, 6, 7, 8, 10, 11, 12]. Cytokines such as VEGF, IL‐1β, PDGF, and FGF promote tumor formation, and IFNs, IL‐2, IL‐12, and IL‐15 inhibit tumor growth [3, 6, 7, 8, 10, 11, 12]. The role of TGF‐β, TNF‐α, IL‐10, IL‐6, and chemokines is more complex, as they may promote or inhibit cancer in a context‐dependent manner [3, 6, 7, 8, 10, 11, 12, 13]. Targeting abnormal cytokine signaling pathways for cancer therapy has shown promising results [7, 9, 12]. Inhibitors of TNF‐α, IL‐6, CCL5, and CXCR4 signaling are approved for clinical use [7, 9, 12]. While molecules targeting TGF‐β, FGF, VEGF, and IL‐10 signaling and their receptors are under advanced clinical evaluation [7, 9, 12].

## Circular RNAs

**Fig. 2:**
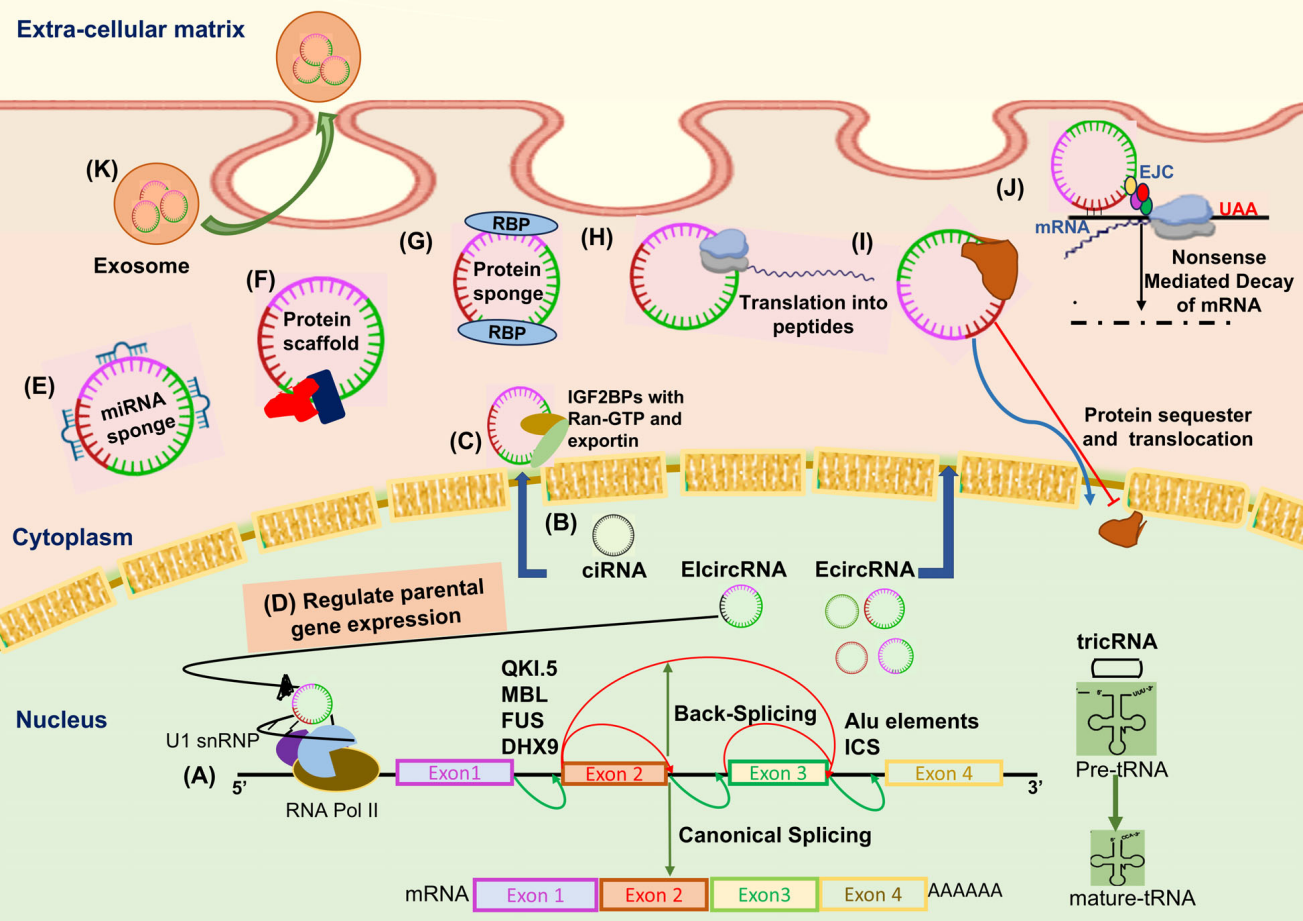
Biogenesis and mechanism of action of circRNAs: (A) CircRNAs are formed through back‐splicing (red arrows), where a downstream splice donor joins an upstream splice acceptor (lacking free 3′OH group), often facilitated or antagonized by intronic complementary sequences or RNA‐binding proteins like QKI, MBL, Fus, and DHX9. (B) Based on the exons and introns orientation, circRNAs are classified into four subtypes: exonic circRNAs (ecircRNAs), circular intronic RNAs (ciRNAs), exonic–intronic circRNAs (EIcircRNAs), and tRNA intronic circRNAs (tricRNAs). (C) The circRNAs are exported from the nucleus to the cytoplasm through the nuclear pore complex with the help of proteins such as IGF2BPs and exportin. (D) In the nucleus, circRNAs can form a complex with U1snRNP that binds with RNA Pol II to regulate the transcription, splicing, and expression of parental genes. (E) In the cytoplasm, circRNAs bind to miRNAs and sequester them to stabilize the expression of their target mRNAs. (F) CircRNAs bind to multiple proteins and act as a scaffold. (G) CircRNAs bind to RBPs and act as protein sponges. (H) Some circRNAs are translated into peptides through IRES‐dependent mechanisms. (I) CircRNAs also sequester proteins to alter their localization or function. (J) CircRNAs can promote nonsense‐mediated decay (NMD) to regulate mRNA stability. (K) CircRNAs can be encapsulated in exosomes and released into the ECM.

CircRNAs are single‐stranded, covalently closed RNA molecules lacking free ends; this endows them with higher stability than mRNAs due to resistance to exonuclease‐mediated degradation [14, 15, 16, 17, 18, 19, 20, 21]. CircRNAs are synthesized by a non‐canonical alternative RNA splicing event known as back‐splicing, where a downstream splice donor (5′ splice site) joins with an upstream splice acceptor (3′ splice site) to create a circular structure featuring a 3′–5′ phosphodiester bond at the back‐splicing junction (Fig. 2) [14, 15, 16, 17, 18, 19, 20, 21, 22]. CircRNA synthesis by back‐splicing may occur co‐transcriptionally or post‐transcriptionally and is favored by the rapid elongation rate of RNA Polymerase II [14, 15, 16, 17, 18, 21, 22, 23]. The biogenesis of circRNAs is facilitated by cis‐elements (e.g., inverted‐repeat Alu elements) and/or trans‐elements (e.g., QKI); the detailed biogenesis of circRNAs is discussed elsewhere (Fig. 2) [16, 23, 24, 25, 26, 27, 28, 29]. More than a million circRNAs have been discovered in human cells through RNA sequencing [14, 20, 30]. However, only a few hundred have been characterized for their physiological functions [30]. Increasing evidence suggests that most circRNAs are non‐coding and regulate cellular processes such as splicing, transcription, epigenetic alterations, and mRNA stability using diverse mechanisms (Fig. 2) [31, 32, 33, 34, 35, 36, 37, 38]. Mechanistically, non‐coding circRNAs (a) bind and sequester miRNAs and proteins, (b) serve as scaffolds for protein complexes, and (c) interact with mRNAs to regulate RNA stability and other cellular processes (Fig. 2) [31, 32, 33, 34, 35, 36, 37, 38, 39]. Interestingly, some circRNAs associate with polyribosomes and are translated into proteins due to the presence of open reading frames (ORFs), internal ribosome entry sites (IRES), and N6‐methyladenosine (m6A) modification [34, 40, 41, 42]. CircRNAs that code for proteins regulate various pathophysiological conditions, including cancer [34]. For example, circZNF609, which regulates myogenesis, is associated with polyribosomes and produces a 250 amino‐acid protein through splicing‐dependent and IRES‐mediated translation [43]. CircPPP1R12A codes for a 73 amino‐acid long polypeptide (circPPP1R12A‐73aa), which drives colon cancer growth and metastasis via Hippo‐YAP signaling [44].

**Fig. 3:**
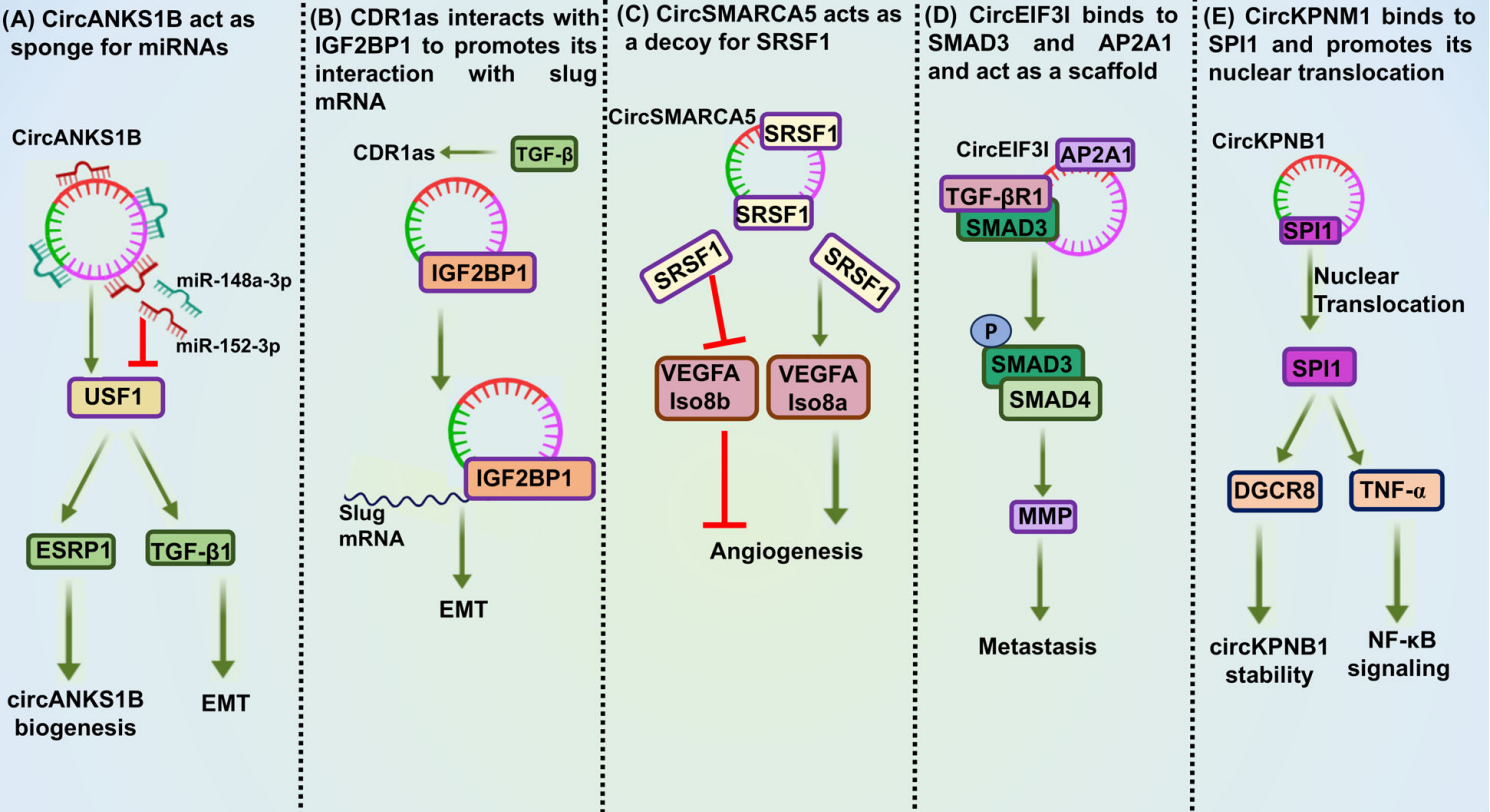
Mechanism of action of circRNAs in regulating cytokine signaling (A) circANKS1B counters repression of USF1 by binding and sponging miR‐148a‐3p and miR‐152‐3p. USF1 upregulates ESRP1 and TGF‐β1 expression. TGF‐β1 activates the downstream SMAD signaling to induce EMT, and ESRP1 promotes circANKS1B biogenesis. (B) TGF‐β induces CDR1as expression; it directly interacts with IGF2BP1 to stabilize Slug mRNA, enhancing its expression to induce EMT. (C) circSMARCA5 acts as a decoy for SRSF1, modulating VEGFA splicing to favor the anti‐angiogenic isoform (Iso8b) over the pro‐angiogenic isoform (Iso8a). (D) circEIF3I acts as a molecular scaffold and forms a ternary complex with SMAD3 and AP2A1, facilitating SMAD3 recruitment in early endosomes. This complex strengthens SMAD3 and TGF‐βRI interaction and enhances the SMAD3 phosphorylation to activate downstream targets like matrix metalloproteinases (MMPs). (E) circKPNB1 interacts with SPI1 to promote its nuclear translocation. SPI1 promotes the transcription of DGCR8 and TNF‐α, influencing circKPNB1 stability and activating NF‐κB signaling. Activating effects (green), inhibitory effects (red).

CircRNAs regulate all aspects of cancer pathogenesis by modulating gene expression, and numerous circRNAs show aberrant expression in cancer tissues [18, 22, 33, 45, 46, 47]. Their intrinsic tissue‐specific expression patterns and exceptional molecular stability make them compelling candidates for developing prognostic and diagnostic biomarkers [14, 47, 48]. CDR1as is one of the most extensively studied circRNAs and has the potential to serve as a prognostic and diagnostic biomarker in colon, lung, and breast carcinomas [49, 50, 51, 52]. CircRNAs are secreted in the exosomes and present in body fluids, making them a suitable candidate as biomarkers for non‐invasive methods of disease diagnosis [53]. CircRNA biogenesis is altered by cytokine signaling, and circRNAs regulate cytokine signaling by altering gene expression using multiple mechanisms (Fig. 3) [22, 28, 33, 54, 55, 56, 57, 58, 59, 60]. Here, we summarize the role of circRNAs in regulating cytokine networks involved in cancer.

## Role of circRNAs in regulating TGF‐β signaling in cancer

The TGF‐β signaling comprises TGF‐β1, TGF‐β2, and TGF‐β3 ligands, which signal via serine/threonine kinase TβRI and TβRII receptors to activate SMAD proteins and other non‐canonical mediators of the TGF‐β pathway [61]. TGF‐β plays dual roles during cancer development by suppressing tumor growth in the initial stages and accelerating tumor growth in the later stages of carcinogenesis [8, 61]. Interestingly, TGF‐β regulates circRNA biogenesis during EMT by activating RNA‐binding protein (RBP) QKI [28]. The role of circRNAs in regulating the TGF‐β signaling is well explored [61].

**Fig. 4:**
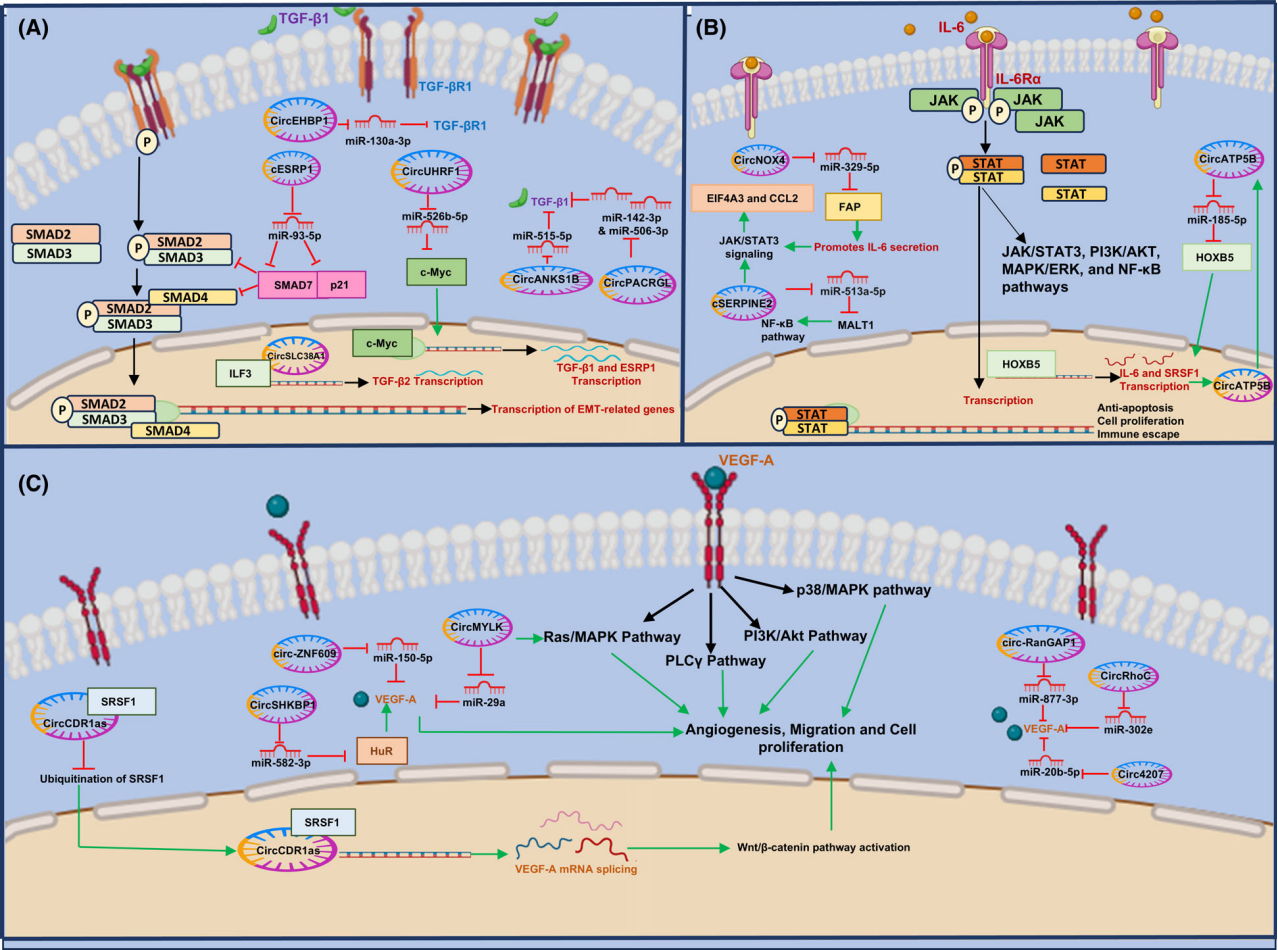
CircRNAs regulate cytokine signaling components and pathways. (A) CircRNAs target the ligands, receptors, and effector proteins in the TGF‐β pathway and modulate EMT. (B) CircRNAs regulate IL‐6 secretion and influence NF‐κB and JAK/STAT signaling cascade, leading to the regulation of apoptosis, cell proliferation, and immune escape. (C) CircRNAs promote VEGFA expression and splicing, activating Ras/MAPK, PI3K/Akt, and PLCγ pathways, which drive angiogenesis, migration, and tumor progression. Activating effects (green), inhibitory effects (red) signaling (black).

Many circRNAs regulate the expression of TGF‐β ligands by acting as miRNA sponges and/or interacting with proteins (Table 1 and Fig. 3) [55, 62, 63, 64, 65, 66, 67]. CircANKS1B promotes TGF‐β1 expression and is upregulated in triple‐negative breast cancer (TNBC) and oral squamous cell carcinoma (OSCC) [55, 62]. In TNBC, it promotes TGF‐β1 transcription and EMT by enhancing the expression of transcription factor USF1 by sponging miR‐148a‐3p and miR‐152‐3p (Fig. 3) [55]. Upregulation of USF1 increases the expression of splicing factor ESRP1, which promotes circANKS1B biogenesis (Fig. 3) [55]. CircANKS1B increases TGF‐β1 expression in OSCC by sponging miR‐515‐5p to promote EMT and cisplatin resistance (Figs 3 and 4) [62]. CircUHRF1 expression is upregulated in OSCC and promotes TGF‐β1 expression [63]. CircUHRF1 acts as a sponge for miR‐526b‐5p to increase c‐Myc expression, which promotes transcription of TGF‐β1 and ESRP1 (Fig. 4) [63]. ESRP1 further accelerates the biogenesis of circUHRF1, thus forming a circUHRF1/miR‐526b‐5p/c‐Myc/TGF‐β1/ESRP1 feedback loop to promote TGF‐β1 induced EMT [63]. CircPACRGL increases TGF‐β1 expression by sponging miR‐142‐3p and miR‐506‐3p in colorectal carcinoma (CRC) (Fig. 4) [64]. Tumor‐derived exosomes stimulate circPACRGL expression to increase the migration, invasion, and differentiation of N1 neutrophils to pro‐tumorigenic N2 neutrophils [64].

**Table 1:** CircRNAs associated with cytokine signaling in cancer.

CircRNA	Cancer	Expression levels (upregulated/downregulated)	Type of regulation	Mechanism of action	Target (interacting proteins/miRNAs)	References
Circular RNAs involved in regulating TGF‐β signaling						
CircANKS1B	TNBC	Upregulated in TNBC tissues when compared to adjacent normal tissues and MDA‐MB‐231, MDA‐MB‐468 and BT54 cells compared to breast epithelial cell line (MCF10A)	Positively regulates TGF‐β/Smad signaling	It promotes TGF‐β induced EMT by sponging miRNAs which increases USF1, resulting in transcriptional activation of TGF‐β1. It also upregulates ESRP1 to promote circANKS1B biogenesis	miR‐148a‐3p and miR‐152‐3p	[55]
CircANKS1B	OSCC	Upregulated in OSCC cells (CAL27, SCC9, and SCC090) when compared with normal oral keratinocyte cell (HOK)	Positively regulates TGF‐β signaling	It acts as miRNA sponge to increase TGF‐β1 expression, which enhances EMT and cisplatin resistance in OSCC	miR‐515‐5p	[62]
CircUHRF1	OSCC	Upregulated in OSCC tissues and SCC25, CAL27, SCC15, and TSCCa cells when compared with normal oral keratinocyte cell (HOK)	Positively regulates TGF‐β signaling	It transcriptionally upregulates TGF‐β1 and ESRP1 by upregulating c‐Myc by acting as miRNA sponge to enhance EMT. ESRP1 also accelerates the biogenesis and circularization of circUHRF1	miR‐526b‐5p	[63]
CircPACRGL	CRC	Upregulated in exosomes of HCT116 and SW480	Positively regulates TGF‐β signaling	It acts as a miRNA sponge to increase TGF‐β1 expression to promote migration, invasion, and N1 to N2 neutrophils differentiation	miR‐142‐3p/miR‐506‐3p	[64]
Circ‐DOCK5	ESCC	Downregulated in ESCC clinical tissues and TE1, KYSE30, KYSE150, and KYSE17 cells compared to normal esophageal tissues	Negatively regulates TGF‐β signaling	It inhibits TGF‐β2 expression and secretion by acting as miRNA sponge to suppress EMT and ZEB1 expression	miR‐627‐3p	[65]
CircCOG2	CRC	Upregulated in CRC tissues and DLD1, SW480, and HCT8 cell lines when compared with normal tissues	Positively regulates TGF‐β/Smad signaling	It activates the TGF‐β2/SMAD3 pathway and EMT via acting as miRNA sponge to promote TGF‐β2 expression	miR‐1305	[66]
CircSLC38A1	BLCA	Upregulated in bladder cancer tissues when compared with normal tissues and J82, T24, RT4, SW780, and 5637 cell line compared to normal uroepithelial cell line SV‐HUC‐1	Positively regulates TGF‐β/Smad signaling	It promotes metastasis and invasion by interacting with ILF3 protein and modulating the ubiquitination process, forming a circSLC38A1‐ILF3 complex that targets the TGF‐β2, thus increasing its expression	ILF3	[67]
CircEHBP1	BLCA	Upregulated in metastatic lymph nodes of BLCA when compared with primary tissues, and UM‐UC‐3, T24, and 5637 cell lines when compared with the normal uroepithelial cell line SV‐HUC‐1	Positively regulates TGF‐β/Smad signaling	It upregulates TGF‐BR1 expression by acting as miRNA sponge, thereby activating the TGF‐β/SMAD3 signaling pathway. This increases the secretion of VEGF‐D to promote lymphangiogenesis and lymphatic metastasis	miR‐130a‐3p	[68]
CircEIF3I	PDAC	Upregulated in PDAC tissues when compared with normal tissues	Positively regulates TGF/Smad signaling	It interacts with the MH2 domain of SMAD3, increasing SMAD3 phosphorylation and MMPs' expression and activity, which promotes migration, invasion, and metastasis. It directly binds with AP2A1, promoting circEIF3I‐bound SMAD3 recruitment to TGFβRI on early endosomes	SMAD3 and AP2A1	[56]
CircPTEN1	CRC	Downregulated in CRC tissues when compared with normal adjacent tissues	Negatively regulates TGF‐β/Smad signaling	It disrupts the TGF‐β/Smad signaling and inhibits the TGF‐β‐mediated EMT by binding to the MH2 domain of Smad4, reducing the formation and translocation of Smad complexes to the nucleus	Smad4	[69]
cESRP1	SCLC	Downregulated in patient‐derived cells that are chemoresistant when compared with patient‐derived cells that are chemosensitive, and H69AR (chemoresistant) cells when compared to H69 chemosensitive cells	Negatively regulates TGF‐β signaling	Negatively regulates TGF‐β mediated EMT by acting as miRNA sponge that promotes SMAD7 and p21 expression	miR‐93‐5p	[70]
CircCDR1as	CC	Upregulated after TGF‐β induction in Siha cell line	Positively regulates TGF‐β signaling	It positively regulates TGF‐β mediated EMT by interacting with IGF2BP1 to increase the stability of Slug	IGF2BP1	[57]
Circ‐AKT1	CC	Upregulated in CC tumor samples when compared with the non‐tumor samples. It is upregulated upon TGF‐β treatment in Siha cells	Effector of TGF‐β signaling	It acts as miRNA sponge to upregulate the expression of AKT1, which promotes tumor growth	miR‐942‐5p	[71]
CircRYK	GBM	Upregulated upon TGF‐β treatment in GBM primary cells. When compared with normal human astrocytes, it is upregulated in U87, U118, U251, T98, LN229 and pGBM‐1 human glioblastoma primary cell line	Effector and positively regulates TGF‐β signaling	It promotes TGF‐β mediated EMT, stemness, and the growth of GBM by enhancing the production and stability of VLDLR (a low‐density lipoprotein receptor) mRNA by acting as miRNA sponge and also increases the interaction of VLDLR with Hur protein	miR‐330‐5p	[72]
Circ6834	NSCLC	Downregulated in NSCLC tissues when compared with non‐cancerous tissues and A549, H1299, and PC9 cell lines when compared with bronchial epithelial cells (HBE)	Negatively regulates TGF‐β/Smad signaling pathway	It inactivates the TGF‐β/Smad signaling pathway by acting as miRNA sponge that upregulates TXNIP and also binds and inhibits the stability of AHNAK, a key regulator of TGF‐β/Smad signaling, by increasing TRIM25‐mediated ubiquitination and degradation	AHNAK, miR‐873‐5p	[73]
CircPTK2	NSCLC	Downregulated in NSCLC tissues when compared to non‐cancerous tissues and A549, H1299, H1650, SPC‐A1, and Calu3 cell lines when compared to human lung normal epithelial cells	Effector and negatively regulates TGF‐β signaling and TGF‐β induced EMT	It inhibits TGF‐β‐induced EMT and cell invasion by acting as miRNA sponge which targets TIF1γ (a negative regulator of TGF‐β/Smad signaling)	miR‐429/miR‐200b‐3p	[74]
Circular RNAs involved in regulating IL signaling						
CircNOX4	NSCLC	Upregulated in CAFs when compared to normal fibroblasts from human NSCLC tissues and adjacent normal tissues and A549, PC9, H226, and H1581 cell lines when compared with human bronchial epithelial cells (BEAS‐2B)	Positively regulates IL‐6	It acts as miRNA sponge to upregulate FAP, which induces IL‐6. It leads to fibroblast activation, increased proliferation, migration, and tumor growth	miR‐329‐5p	[78]
cSERPINE	BC	Upregulated in breast cancer tissues when compared to normal adjacent tissues and MCF‐7 and MDA‐MB cell lines when compared to normal breast cell line (MCF‐10A)	Positively regulates IL‐6	It activates the NF‐B pathway to increase the secretion of IL‐6 in TAMS in breast cancer. It functions by acting as miRNA sponge in TAMs to upregulate the MALT1 expression, a transducer of NF‐B signaling	miR‐513a‐5p	[79]
CircATP5B	Glioma	Upregulated in the glioma tissues when compared with adjacent brain tissue samples	Positively regulates IL‐6	It acts as miRNA sponge that upregulates HOXB5 expression and transcriptionally regulates IL6 expression, promoting the proliferation of GSC via JAK2/STAT3 signaling. Also, SRSF1 binds to and promotes circATP5B expression	miR‐185‐5p/SRSF1	[80]
CircNFIX	OC	Upregulated in OC tumor tissues when compared with adjacent tissues and multiple OC cell lines (PEO1, 3AO, SKOV3, OVCAR3, CAOV3, and A2780) when compared with normal ovarian epithelial cell line (HOSEPiCs)	Positively regulates IL‐6R	m6A activated‐circNFIX promotes immune escape by positively regulating IL6R. It acts as miRNA sponge to increase IL‐6R, thus activating the JAK/STAT signaling and increasing PD‐L1 expression	miR‐647	[81]
cGGNBP2	ICC	Upregulated in ICC tumor when compared with non‐tumor. It is upregulated upon IL6 treatment in ICC cell lines (HuccT1, RBE)	Positively regulates IL‐6	It encodes for 184aa protein and directly interacts with STAT3, enhancing the phosphorylation of STAT3. This increases the growth and migration of ICC cells. IL‐6/cGGNBP2‐184aa/STAT3 forms a positive feedback loop to sustain constitutive activation of IL‐6/STAT3 signaling	STAT3	[82]
CircPOLQ	CC	Upregulated in CRC tissues when compared with normal tissues and HCT116, LoVo, HT‐29, SW620 cell lines when compared with normal cell line (FHC)	Positively regulates IL‐10	It increases IL10 expression and activates the IL‐10/STAT3 axis by acting as miRNA sponge to promote M2 macrophage polarization and metastasis	miR‐379‐3p	[83]
CircLOC729852	BLCA	Upregulated in BLCA tissues when compared with normal tissues and T24, UM‐UC‐3 cell lines when compared with normal cell line (SV‐HUC‐1)	Positively regulates IL‐10	It increases the proliferation, migration, and EMT by acting as miRNA sponge, which can upregulate IL‐10 expression. CircLOC729852/miR‐769‐5p/IL‐10 axis activates JAK2/STAT3 signaling that modulates autophagy and promotes the recruitment and M2 polarization of TAMs	miR‐769‐5p	[84]
CircMERTK	CRC	Upregulated in HCT116 TAM‐like cells and HT29 TAM‐like cells compared with control group. It is also upregulated in CRC tissues (TAMS of CRC) than in matched normal tissues	Positively regulates IL‐10	It acts as miRNA sponge to upregulate IL‐10 expression, promoting the apoptosis of CD8^+^ T cells and impacting the immunosuppressive activity of TAM‐like cells cell	miR‐125a‐3p	[85]
Circular RNAs involved in regulating TNF‐α signaling						
CircKPNB1	GBM	Upregulated in glioma tissues when compared to normal brain tissues	Positively regulates TNF‐α signaling	It interacts with SPI1 and regulates the protein stability and nuclear translocation of SPI1. It acts in a positive feedback regulatory loop where SPI1 transcriptionally upregulates TNF‐α and DGCR8. DGCR8 binds to circKPNB1 to maintain its stability, promoting the upregulation and secretion of TNF‐α, activating NF‐κB signaling. It promotes the proliferation, migration, stemness, and neurosphere formation abilities of GSCs	SPI1	[58]
CircDOCK1	OSCC	Downregulated in apoptosis model compared with the negative group. It is upregulated in the OSCC tissue compared with the para‐carcinoma tissue	Effector of TNF‐α signaling	It acts as miRNA sponge which targets BIRC3, resulting in its increased expression and decreased apoptosis	miR‐196a‐5p	[86]
Circular RNAs involved in regulating Chemokine signaling						
Circ_0002483	LUAD	Upregulated in pair‐matched LUAC tissue samples as compared with the non‐cancerous tissues	Positively regulates chemokine signaling	It promotes proliferation, migration invasion, and tumor growth by positively regulating CCL4 via sponging miRNA and negatively regulating its expression	miR‐125a‐3p	[88]
Circ_0003410	HCC	Upregulated in HCC specimen when compared with the non‐cancerous tissues and Hep1, HepG2, Huh7, and SMMC772 cell lines when compared with the normal cell line (LO2)	Positively regulates chemokine signaling	It positively regulates the expression of *CCL5* by acting as miRNA sponge to recruit macrophages, resulting in increased proliferation and migration	miR‐139‐3p	[89]
CircETFA	HCC	Upregulated in tumor tissue and plasma of HCC patients when compared to healthy controls and YY8103 and Hep3B cell lines when compared to normal liver cell lines (HL7702)	Positively regulates chemokine signaling	It acts as miRNA sponge, which targets CCL5 and also recruits EIF4A3 to extend the half‐life of CCL5, thus increasing the expression of CCL5	miR‐612	[90]
CircCYP24A1	ESCC	Upregulated in ESCC tissue group when compared with normal tissues and have a different endogenous expression in ESCC cell lines	Positively regulates chemokine signaling	It binds with PKM2 and activates the NF‐κB pathway, which promotes the secretion of CCL5 and promotes proliferation, migration, invasion, and clone formation, as well as tumor growth	PKM2	[91]
CircMAPK1	LUAD	Downregulated in LUAD tissues when compared with the adjacent normal tissues and A549, PC9, H1975, and H358 cell lines when compared with normal human bronchial epithelial cell line (BEAS‐2B)	Positively regulates chemokine signaling	It binds to IGF2BP1 to retain the stability of CCL5 mRNA, a chemokine that recruits CD8^+^ T cells	IGF2BP1	[92]
CircSMARCC1	PC	Upregulated in plasma samples of patients with benign prostatic hyperplasia (BPH) and PCa when compared with the control and PC‐3, DU145, 22Rv1, C4–2, and LNCaP cell lines when compared with the normal prostate epithelial cell line	Positively regulates chemokine signaling	It positively regulates CC‐chemokine ligand 20 (CCL20) and activates the PI3K‐Akt signaling pathway by acting as miRNA sponge increasing proliferation and EMT. It is also positively associated with colonization of CD68^+^/CD163^+^/CD206^+^ TAMs in tumor microenvironment and facilitates the expression of CD163 in macrophages through the CCL20‐CCR6 axis, inducing TAMs infiltration and M2 polarization	miR‐1322	[93]
Circ_0004140	LUAD	Upregulated in LUAD clinical samples when compared with normal adjacent tissue and A549, SPCA‐1, NCI‐H446, and NCI‐H292 cell lines when compared with normal cell line (BEAS‐2B)	Positively regulates chemokine signaling	It increases cell proliferation, migration, and resistance to anti‐PD‐1 immunotherapy by acting as miRNA sponge to increase CCL22	miR‐1184	[94]
circDHTKD1	BLCA	Upregulated in BLCA tissues when compared with normal tissues and 5637, T24, and UMUC3 cell lines when compared with urothelial cell line SV‐HUC‐1	Positively regulates chemokine signaling	It acts as miRNA sponge and antagonizes the repression of miRNA on CXCL5, which facilitates lymphangiogenesis and LN metastasis	miR‐149‐5p	[95]
Circ_0073453	GC	Upregulated in Gastric cancer‐associated mesenchymal stem cells (GC‐MSCs) when compared with bone marrow‐derived mesenchymal stem cells (BM‐MSCs)	Positively regulates chemokine signaling	It acts as miRNA sponge, which inhibits IL‐8, thus increasing IL‐8 expression and secretion, promoting metastasis. It also enhances PD‐L1 expression to resist cytotoxic CD8^+^ T cell‐killing by modulating IL‐8 secretion by GC‐MSCs	miR‐146a‐5p	[96]
Circ_0007432	NSCLC	Upregulated in NSCLC tumor tissues when compared with para‐carcinoma tissues and NSCLC cell lines (PC‐9, Calu‐3, H1975, A549, and H358) when compared with human bronchial epithelial cells (BEAS‐2B)	Positively regulates chemokine signaling	It acts as a protein scaffold that interacts with SRSF1 protein and recruits it to KLF12 mRNA, thus increasing the KLF12 expression. KLF12 facilitates IL‐8 expression and promotes IL‐8 release, resulting in increased proliferation, migration, and invasion	SRSF1	[97]
Circ_0000515	BC	Upregulated in BC tissues when compared with non‐tumor tissues and MDA‐MB‐231, SUM‐159, MCF‐7, SK‐BR‐3, and MDA‐MB‐157 cell lines when compared with normal breast epithelial cell line (MCF10A)	Positively regulates chemokine signaling	It acts as miRNA sponge, which targets CXCL10, thus increasing its expression, resulting in cell cycle progression, increased proliferative, invasive, and pro‐angiogenetic abilities, and increased inflammatory response	miR‐296‐5p	[98]
CircDLG1	GC	Upregulated in distant metastatic lesions and primary gastric cancer tissues resistant to anti‐PD‐1 therapy in a larger cohort of patients	Positively regulates chemokine signaling	It acts as miRNA sponge to increase CXCL12, promoting proliferation, migration, invasion, immune evasion, and resistance to anti‐PD‐1‐based therapy	miR‐141‐3p	[99]
Circ_0020710	Melanoma	Upregulated in melanoma tissues when compared to paired normal tissues and benign nevi tissues. It is upregulated in melanoma cell lines compared with HaCaT, a normal epidermal cell line	Positively regulates chemokine signaling	It promotes cell proliferation, migration, and invasion by upregulating the CXCL12 expression via sponging miRNA	miR‐370‐3p	[100]
CircFGFR1	NSCLC	Upregulated in NSCLC tumor tissues and when compared to the matched adjacent non‐tumor lung tissues. It has different endogenous expression in NSCLC cell line (NCI‐H358, NCI‐H1299, A549, HCC827, NCI‐H1650, NCI‐H838, and NCI‐H292)	Positively regulates chemokine signaling	It promotes proliferation, migration, invasion, and immune evasion by acting as miRNA sponge to upregulate the expression of CXCR4 and also provides resistance to anti‐programmed cell death 1 (PD‐1)‐based therapy	miR‐381‐3p	[101]
CircBACH2	TNBC	Upregulated in TNBC cancerous tissues compared with adjacent normal tissue and MDA‐MB‐231 and BT‐549 cell lines compared with normal mammary gland cell line (MCF‐10A) and cell lines of other subtypes of breast cancer (MCF‐7 and BT‐474)	Positively regulates chemokine signaling	It promotes cell proliferation, migration, and invasion by sponging miRNAs, which increases CXCR4 expression	miR‐186‐5p and miR‐548c‐3p	[102]
Circular RNAs involved in regulating VEGF signaling						
Circ‐ZNF609	ESCC	Upregulated in hypoxic cultured ESCC cells when compared with the normoxic cultured ESCC cells	Positively regulates VEGF signaling	It positively regulates VEGFA by sponging miR‐150‐5p that targets VEGFA and also interacts with HuR to inhibit its binding with ZO‐1, Claudin‐1, and Occludin mRNAs, affecting their translation	miR‐150‐5p and Hur	[104]
Circ4207	CRC	Upregulated in CRC tissues when compared with normal adjacent tissues and SW480, Caco2, HCT116, Lovo, SW620 cell lines when compared with human normal colon epithelial cells (NCM460)	Positively regulates VEGF signaling	It promotes proliferation and invasion and facilitates vascular mimicry formation via sponging miRNA that targets VEGFA and increases its expression	miR‐20b‐5p	[105]
Circ‐RanGAP1	GC	Upregulated in GC tissues and plasma exosomes when compared with normal tissue. It is upregulated in stage III GC tissues compared with stage I‐II GC tissues	Positively regulates VEGF signaling	It acts as miRNA sponge to increase the expression of VEGFA to promote migration and invasion	miR‐877‐3p	[106]
CircMYLK	BLCA	Upregulated in BLCA tissues compared with the adjacent non‐tumor tissues and T24, EJ, BIU‐87, 5673 cell lines compared with the normal bladder epithelial cell line (SV‐HUC‐1)	Positively regulates VEGF signaling	It promotes the expression of VEGFA by acting as miRNA sponge which promotes tumor growth and metastasis	miR‐29a	[107]
CircRhoC	OC	Upregulated in ovarian cancer tissues when compared to normal ovarian tissues	Positively regulates VEGF signaling	It acts as miRNA sponge to promote VEGFA expression, which promotes growth, angiogenesis, and lymphangiogenesis	miR‐302e	[108]
Circ_0059914	Glioma	Upregulated in glioma cells (U251, U87MG, and A172) when compared to normal glial HA cells	Positively regulates VEGF signaling	It promotes proliferation, migration, invasion, EMT, and angiogenesis by sponging miRNA which targets VEGFA, thus increasing its expression	miR‐1249	[109]
CircCDR1as	Lung cancer	Upregulated in PM_2.5_‐induced lung cancer cells (H1299, A549 and H460)	Positively regulates VEGF signaling	It inhibits apoptosis and promotes malignant behavior in lung cancer cells by binding to the SRSF1 protein to affect its function, influencing the splicing of VEGFA	SRSF1	[51]
CircPAK2	GC	Upregulated in GC tissues and metastatic lymph nodes when compared to normal adjacent tissues. It is upregulated in GC cell lines (HGC‐27, AGS, MKN45, MKN‐74) when compared to normal cell line (GES‐1)	Positively regulates VEGF signaling	It interacts with IGF2BPs, forming circPAK2/IGF2BPs/VEGFA complex, and stabilizes VEGFA, promoting migration, invasion, EMT, angiogenesis, lymphangiogenesis, and metastasis	IGF2BPs	[110]
CircSMARCA5	GBM	Downregulated in GBM biopsies when compared with unaffected brain parenchyma (UC)	Negatively regulates VEGF signaling	It acts as molecular decoy by directly interacting with SRSF1, thereby modulating the balance between pro‐ and anti‐angiogenic isoforms of VEGFA pre‐mRNA	SRSF1	[59, 60]
CircSHKBP1	GC	Upregulated in GC tissues when compared with the matched normal tissues and BGC823, HGC27, AGS, and MGC803 cell lines when compared with the normal gastric epithelial cell line (GES1)	Positively regulates VEGF signaling	It promotes proliferation, migration, and invasion by sponging miRNA which alleviates the expression of Hur and stabilizes the VEGF mRNA. It also interacts with HS90 to prevent its degradation from STUB1	miR‐582‐3p/HS90	[111]
CircNFIB1	PDAC	Downregulated in PDAC tissues when compared with corresponding normal adjacent tissues and PANC1, Capan‐2, SW1990 cell line when compared with human pancreatic ductal endothelial cells (HPDE)	Negatively regulates VEGF signaling	It suppresses LN metastasis by serving as miRNA sponge upregulating the expression of its target gene PIK3R1, which inhibits the PI3K/Akt pathway, thus downregulating VEGF‐C expression	miR‐486‐5p	[112]
Circular RNAs involved in regulating FGF signaling						
CircFGFR1int2	PCa	Upregulated in PCa tissues when compared to the normal prostate tissues and 22Rv1, LNCap, PC‐3, and DU145 cell lines when compared to normal cell line (RWPE‐1)	Positively regulates FGF	CircFGFR1^int2^ recruits the transcription activators P65 and FUS enhancing *FGFR1* expression at both the transcription and the post‐transcription levels. It also suppresses miRNA which targets *FGFR1* and increases its expression	P65 and FUS; miR‐4687‐5p	[115]
CircRNA_103809	HCC	Upregulated in HCC tissue samples when compared with normal adjacent tissue samples and MHCC97L, Huh7, SK‐HEP‐1, Hep3B, HCCLM3 cell lines when compared with human normal hepatocyte (LO2)	Positively regulates FGF	It increases the proliferation, cycle progression, and migration by sponging miRNA to increase FGFR1 expression	miR‐377‐3p	[116]
CircRAPGEF5	Papillary thyroid	Upregulated in PTC tissues and BCPAP, KTC‐1, and K1 cell lines compared to normal PTC cells (Nthy‐ori 3‐1)	Positively regulates FGF	CircRAPGEF5 increases cell proliferation, migration, and invasion and increases the expression of FGFR1 by sponging miRNA that targets FGFR1	miR‐198	[117]
CircUVRAG	BLCA	Upregulated in BLCA tissues when compared with control groups and EJ, T24, J82, UM‐UC‐3, TCC, and RT‐4 cell lines when compared with normal urothelial cells (SV‐HUC cells)	Positively regulates FGF	circUVRAG promotes the proliferation and metastasis abilities of BLCA cells by sponging miRNA to increase FGFR2 expression	miR‐223	[118]
Circ_0068871	BLCA	Upregulated in BLCA tissues when compared with normal adjacent tissues and T24, UMUC3, EJ, and J82 cell lines when compared with normal urothelial cells (SV‐HUC cells)	Positively regulates FGF	It promotes proliferation, migration and inhibits apoptosis by upregulating FGFR3 expression, and activates STAT3 by acting as miRNA sponge	miR‐181a‐5p	[119]
Circular RNAs involved in regulating PDGF signaling						
CircCDK14	Glioma	Downregulated in glioma tissues compared to non‐tumor brain tissues and U251, U87, and SF126 cell lines compared to normal brain glial cells (HEB)	Positively regulates PDGF	It accelerates PDGFRA expression by sequestering miRNA which leads to increased invasion and also reduces the glioma cells' sensitivity to ferroptosis	miR‐3938	[121]
CircMETRN	GBM	Upregulated in low‐dose radiation (LDR) group when compared to the negative control (NC) group and high‐dose radiation (HDR) group	Positively regulates PDGF	It acts as miRNA sponge that targets GRB14 which increases PDGFRα levels	miR‐4709‐3p	[122]
CircCHD7	EC	Upregulated in EC tissue samples and cell line (Ishikawa cells and HEC‐1B) when compared with normal endometrium tissue samples	Positively regulates PDGF	It enhances the mRNA stability of PDGFRB by interacting with IGF2BP2 and increases proliferation	IGF2BP2	[123]

CircRNAs also regulate TGF‐β2 expression (Table 1 and Fig. 4) [65, 66, 67]. Circ‐DOCK5 is downregulated in esophageal squamous cell carcinoma (ESCC) tissues, and it sponges miR‐627‐3p, which inhibits TGF‐β2 expression [65]. ZEB1 inhibits the transcription of eIF4A3, which promotes circ‐DOCK5 biogenesis [65]. ZEB1‐mediated circ‐DOCK5 downregulation alters the miR‐627‐3p/TGF‐β2 signaling to promote metastasis [65]. CircCOG2 expression is elevated in CRC, and it promotes EMT by activating the TGF‐β2/SMAD3 pathway via sponging miR‐1305 [66]. CircSLC38A1 activates TGF‐β2 transcription and EMT in bladder cancer (BLCA) [67]. It interacts with ILF3 (RBP) to stabilize ILF3 and form a circSLC38A1‐ILF3 complex, which functions by binding to the promoter of TGF‐β2 (Fig. 4) [67].

CircRNAs also regulate the expression of the TGF‐βRI receptor (Fig. 4) [68]. CircEHBP1 sponges miR‐130a‐3p and prevents the inhibition of to enhance TGF‐β/SMAD3 pathway activation and VEGF‐D secretion in BLCA (Fig. 4) [68].

CircRNAs not only regulate the expression of the ligands and receptors in the TGF‐β pathway but also modulate the activity of its downstream effectors (Table 1 and Fig. 4) [56, 69, 70]. In pancreatic ductal adenocarcinoma (PDAC), circEIF3I acts as a molecular scaffold that interacts with SMAD3 and AP2A1 to form a ternary complex, facilitating the recruitment of SMAD3 in early endosomes (Fig. 3) [56]. This strengthens the SMAD3 and TGF‐βRI interaction to elevate SMAD3 phosphorylation, promoting matrix metalloprotease (MMP) expression (Fig. 3) [56]. CircPTEN1 is a potent tumor suppressor circRNA in CRC whose downregulation in tumor samples correlates with poor patient survival [69]. It directly binds to SMAD4 and disrupts its interaction with SMAD2/3 to suppress their nuclear translocation [69]. This reduces the expression of EMT‐related genes induced by TGF‐β [69]. cESRP1 overexpression sensitizes small‐cell lung cancer (SCLC) cells to chemotherapy by prohibiting the TGF‐β mediated EMT [70]. It rescues the TGF‐β pathway inhibitor SMAD7 and p21 from miR‐93‐5p mediated repression to form a negative feedback loop and inhibit the TGF‐β signaling pathway (Fig. 4) [70].

Interestingly, TGF‐β also regulates the expression and biogenesis of circRNAs to mediate tumor progression [57, 71, 72, 73, 74]. CircCDR1as is one of the most studied circRNAs; TGF‐β induces its expression in cervical cancer (CC) cells [57]. CircCDR1as stabilizes Slug mRNA, a TGF target gene, by interacting with IGF2BP1 (Fig. 3) [57]. Circ‐AKT1 expression is also induced upon TGF‐β in CC cells and tissues [71]. It sponges miR‐942‐5p to increase AKT1 expression, a non‐canonical mediator of TGF‐β signaling to promote EMT and tumor growth [71]. TGF‐β1 upregulates circRYK expression in glioblastoma (GBM) [72]. It interacts with miR‐330‐5p and HuR to promote VLDLR expression and VLDLR mRNA stability, respectively, to increase EMT and GSC maintenance [72]. TGF‐β suppresses the expression of circ6834 and circPTK2 by reducing QKI levels in non‐small cell lung cancer (NSCLC) [73, 74]. Circ6834 sponges miR‐873‐5p to increase the expression of TXNIP, an inhibitor of TGF‐β signaling [73]. It also binds to AHNAK, a positive regulator of TGF‐β signaling, to increase its ubiquitination and degradation by TRIM25, resulting in the inhibition of TGF‐β induced EMT [73]. The expression of CircPTK2 and TIF1γ (an antagonist of TGF‐β signaling) is significantly downregulated in NSCLC cells undergoing TGF‐β induced EMT [74]. CircPTK2 functions as a metastasis suppressor by acting as a sponge of miR‐429/miR‐200b‐3p to promote TIF1γ expression [74].

## Role of circRNAs in regulating IL‐6 and IL‐10 signaling in cancer

Many interleukins, such as IL‐1, IL‐6, IL‐4, IL‐2, and IL‐10, play pivotal roles in tumorigenesis [75]. However, the role of circRNAs has been studied only in the context of IL‐6 and IL‐10 signaling in cancer (Table 1). IL‐6 and IL‐10 are pleiotropic cytokines mediating both pro‐ and anti‐tumorigenic roles during cancer development [75, 76]. IL‐6 and IL‐10 promote tumorigenesis by regulating JAK/STAT3, PI3K/AKT, MAPK/ERK, and NF‐κB pathways to foster proliferation, metastasis, and therapeutic resistance [76, 77]. Conversely, they can enhance anti‐tumor immunity by activating cytotoxic Tcells, triggering acute inflammation, and thereby eliminating malignant cells [76, 77]. IL‐6 activates downstream signaling pathways through the gp130 receptor subunit, which undergoes homodimerization with membrane‐bound IL‐6Rα or IL‐11Rα to initiate classical cis‐signaling or a soluble receptor, resulting in trans‐signaling [75].

CircRNAs regulate the expression of IL‐6 and its receptor IL‐6R (Table 1 and Fig. 4) [78, 79, 80, 81, 82]. In NSCLC, CircNOX4 expression is upregulated in CAFs, creating a pro‐metastatic inflammatory microenvironment by increasing IL‐6 secretion [78]. It binds to miR‐329‐5p, countering its suppressive effect on fibroblast activation protein (FAP) expression, leading to IL‐6 secretion (Fig. 4) [78]. Exosomes from breast cancer (BC) contain cSERPINE2, which shuttles to TAMs to increase IL‐6 secretion and NF‐κB signaling [79]. In TAMs, cSERPINE2 functions by sponging miR‐513a‐5p to upregulate MALT1, a transducer of NF‐κB signaling (Fig. 4) [79]. The secreted IL‐6 activates the JAK/STAT3 pathway in BC cells to increase EIF4A3 and CCL2 levels, which further promotes the biogenesis of cSERPINE in a positive feedback mechanism, resulting in increased invasion, metastasis, and poor patient survival [79]. CircATP5B accelerates IL‐6 transcription to promote the proliferation of glioma stem cells (GSCs) [80]. CircATP5B upregulates HOXB5 expression via sponging miR‐185‐5p [80]. HOXB5 transcriptionally activates both IL‐6 and SRSF1 (Fig. 4) [80]. SRSF1 increases circATP5B expression, forming a SRSF1/circATP5B/miR‐185‐5p/HOXB5 axis to activate JAK/STAT signaling in GSCs [80]. In ovarian cancer (OC), circNFIX sponges miR‐647 to upregulate IL‐6R expression to promote JAK/STAT3 signaling [81]. IGF2B1/2/3 positively regulates circNFIX expression, promoting metastasis, immune escape, and poor patient survival [81]. IL‐6 induces cGGNBP2 expression in intrahepatic cholangiocarcinoma (ICC), which encodes a cGGNBP2‐184aa protein [82]. IL‐6 also rescues the inhibitory effect of DHX9 on cGGNBP2 biogenesis by downregulating its expression [82]. cGGNBP2‐184aa interacts and phosphorylates STAT3 to form a positive feedback loop for sustained IL‐6/STAT3 signaling and metastasis [82].

Currently, very few circRNAs are known to regulate IL‐10 signaling in cancer; their function and mechanism of action are listed in (Table 1) [83, 84, 85].

## Role of circRNA in regulation of TNF‐α signaling in cancer

TNF‐α is a proinflammatory cytokine that activates several signaling cascades, including NF‐κB and MAPK signaling pathways, by interacting with TNF receptors 1 and 2 (TNFR‐1, TNFR‐2) [13]. It mediates dual roles during cancer progression, promoting tumor growth through inflammation, angiogenesis, and immune evasion while also inducing tumor cell death via apoptosis and immune activation [13].

CircRNAs modulate the key signaling molecules in the TNF‐α pathway to modulate inflammation and cell survival (Table 1) [58, 86]. CircKPNB1 increases the self‐renewal of GSCs by activating TNF‐α signaling [58]. Its overexpression promotes invasion, neurosphere formation, and stemness by increasing the protein stability and nuclear translocation of SPI1 (Fig. 3) [58]. SPI1 increases TNF‐α transcription and NF‐κB signaling [58]. SPI1 also forms a positive feedback loop among DGCR8/circKPNB1/SPI1 by upregulating DGCR8 (RBP) to maintain circKPNB1 stability (Fig. 3) [58]. TNF‐α induced OSCC cell death is marked by downregulation of circDOCK1 expression [86]. CircDOCK1 suppresses TNF‐induced cell apoptosis by alleviating the repressive effect of miR‐196a‐5p on BIRC3 (inhibitor of apoptosis) [86].

## Role of circRNAs in regulating chemokine signaling in cancer

Chemokines are divided into CC, CXC, CX_3_C, and XC subfamilies based on the variation in the precise configuration of the two cysteines closest to their N terminus [5, 6, 7, 8]. They play paradoxical roles in carcinogenesis by either promoting tumor progression through immunosuppressive cell recruitment and angiogenesis or facilitating tumor regression by enhancing anti‐tumor immune responses [7, 8, 87]. Chemokines interact with G protein‐coupled receptors (GPCRs) to activate MAPK, PI3K‐AKT, and NF‐κB pathways [7, 8, 87].

Multiple studies have highlighted the role of circRNAs in regulating the expression of chemokine ligands (Table 1) [88, 89, 90, 91, 92, 93, 94, 95, 96, 97, 98, 99, 100]. CCL5 is a pro‐tumorigenic chemokine that promotes tumor growth, metastasis, immune evasion, and angiogenesis by binding to CCR1, CCR3, and CCR5 receptors [5]. Circ_0003410 promotes hepatocellular carcinoma (HCC) proliferation and migration by sponging miR‐139‐3p and upregulating CCL5 expression [89]. CircETFA also increases CCL5 expression in HCC via sponging miR‐612 and prolongs CCL5 mRNA half‐life by recruiting EIF4A3 (RBP) to CCL5 [90]. CircCYP24A1 upregulation in ESCC is associated with poor survival [91]. CircCYP24A1 activates the NF‐κB pathway by binding to PKM2, which promotes the secretion of CCL5 [91]. In contrast to the previous studies, upregulation of CCL5 levels by circMAPK1 inhibits lung adenocarcinoma (LUAD) growth by promoting T‐cell intratumoral infiltration [92]. CircMAPK1 interacts with IGF2BP1 to increase its occupancy on 3′UTR of CCL5 mRNA, increasing its stability and expression in LUAD [92].

CCL20 is a proinflammatory chemokine that promotes proliferation, metastasis, and immune evasion by binding to CCR6 [5]. CircSMARCC1 activates PI3K‐AKT signaling via the CCL20/CCR6 axis in prostate cancer (PC) [93]. It sponges miR‐1322 to upregulate CCL20 expression, accelerating TAM infiltration, M2 macrophage polarization, and metastasis [93]. CCL22 interacts with CCR4 to promote proliferation, immune evasion, and therapy resistance [5]. Circ_0004140 promotes LUAD growth by sponging miR‐1184 to increase CCL22 expression [94]. Circ_0004140 upregulation correlates with cytotoxic T‐lymphocyte (CTL) cell dysfunction and poor patient survival through increased migration and resistance to anti‐PD‐1 immunotherapy [94].

CXCL8/IL‐8 is a proinflammatory chemokine that interacts with CXCR1 and CXCR2 to promote tumorigenesis [5]. Circ_0073453 expression is upregulated in gastric cancer‐mesenchymal stem cells [96]. It enhances CXCL8 expression and secretion by sponging miR‐146a‐5p to promote PD‐L1 expression, immune evasion, and metastasis in GC [96]. Circ_0007432 increases CXCL‐8 expression by binding to SRSF1, stabilizing KLF12 in NSCLC [97]. KLF12 facilitates CXCL8 expression and release by binding to the CXCL8 promoter, increasing migration, invasion, and M2 macrophage polarization [97]. CXCL12 is a homeostatic chemokine that binds to CXCR4 and CXCR7 to promote stemness, immune evasion, metastasis, and angiogenesis [5]. Overexpression of circDLG1 in gastric cancer (GC) enhances CXCL12 expression by sponging miR‐141‐3p to promote stemness, anti‐PD‐1 therapy resistance, and MDSCs infiltration [99]. Upregulation of circ_0020710 in melanoma increases CXCL12 levels by sponging miR‐370‐3p to promote migration, invasion, and anti‐PD‐1 therapy resistance [100]. CXCR4 receptor expression is also modulated by circRNAs (Table 1) [101, 102]. CircFGFR1 enhances CXCR4 expression in NSCLC by sponging miR‐381‐3p to facilitate invasion, immune evasion, anti‐PD‐1 therapy resistance, and poor patient survival [101]. In TNBC, CircBACH2 increases CXCR4 expression by interacting with miR‐186‐5p and miR‐548c‐3p, promoting invasion and metastasis [102].

## Role of circRNAs in regulating VEGF signaling in cancer

The VEGF family of cytokines includes VEGFA/B/C/D and placental growth factor (PIGF), which binds to VEGFR receptors to activate Ras/MAPK, PI3K/Akt, PLCγ, and p38/MAPK pathways [103]. VEGF promotes cancer development by regulating angiogenesis, migration, and neovascularization during carcinogenesis [103].

Multiple circRNAs regulate the expression of VEGF isoforms (Table 1 and Fig. 4) [51, 59, 60, 104, 105, 106, 107, 108, 109, 110, 111, 112]. Hypoxia induces the expression of exosomal circ‐ZNF609 in ESCC [104]. It sponges miR‐150‐5p to increase VEGFA expression, angiogenesis, and metastasis (Fig. 4) [104]. Circ‐ZNF609 also interacts with HuR and blocks its interaction with ZO‐1, Claudin‐1, and Occludin mRNAs, suppressing their expression [104]. Circ4207 alleviates the suppressive effects of miR‐20b‐5p on VEGFA expression in CRC, leading to enhanced invasion, angiogenesis, and tumor growth (Fig. 4) [105]. The upregulation of circ‐RanGAP1 in GC tissues and plasma‐derived exosomes increases VEGFA expression by sponging miR‐877‐3p to enhance tumor growth (Fig. 4) [106]. CircMYLK upregulation promotes EMT and angiogenesis in BLCA by interacting with miR‐29a to activate the VEGFA/VEGFR2 and downstream Ras/ERK signaling pathway (Fig. 4) [107]. CircRhoC promotes OC by sponging miR‐302e to promote VEGFA expression, invasion, and angiogenesis (Fig. 4) [108]. EIF4A3‐induced circ_0059914 upregulates VEGFA by sponging miR‐1249 to increase EMT, angiogenesis, and tumor growth in glioma [109].

CircCDR1as binds to SRSF1 and prevents its ubiquitination and degradation in lung cancer (Fig. 4) [51]. SRSF1 enhances the splicing of VEGFA, causing wnt/β‐catenin pathway activation and invasion (Fig. 4) [51]. Expression of CircPAK2 is upregulated in GC; it binds to IGF2BPs, stabilizing VEGFA mRNA to promote angiogenesis [110]. Downregulation of circSMARCA5 in GBM is associated with poor survival of patients; it directly interacts with the splicing regulator SRSF1, acting as a molecular decoy; this shifts the VEGFA splicing towards the anti‐angiogenic isoform of VEGFA (Iso8b) (Fig. 3) [59, 60]. CircSHKBP1 sponges miR‐582‐3p to increase HUR expression, which in turn stabilizes VEGFA mRNA in GC (Fig. 4) [111]. It also sequesters HSP90 to prevent HSP90 ubiquitination and degradation by STUB1 ubiquitin ligase, leading to increased invasion, angiogenesis, and poor patient survival [111].

## Role of circRNAs in regulating FGF signaling in cancer

FGFs interact with FGF receptors along with heparan sulfate proteoglycans (HSPG) coreceptors to activate Ras/Raf–MEK‐MAPKs, PI3K/Akt, PLCγ, and STAT pathways to increase the motility, invasiveness, and resistance to therapy in cancer [113, 114]. CircRNAs regulate the expression of FGFR during cancer development (Table 1) [115, 116, 117, 118, 119]. CircFGFR1int2 expression is upregulated in PC, and it recruits the transcription activators P65/FUS on the FGFR1 promoter to enhance FGFR1 expression [115]. It also sponges miR‐4687‐5p to suppress its inhibitory effects on FGFR1 mRNA [115]. CircRNA_103809 upregulation correlates with poor survival of HCC patients; it activates the FGFR1/ERK axis by sponging miR‐377‐3p [116]. CircRAPGEF5 promotes FGFR1 expression by sequestering miR‐198 to increase migration, invasion, and tumor growth in papillary thyroid carcinoma [117]. CircUVRAG increased FGFR2 expression by sponging miR‐223 to promote migration, invasion, and metastasis in BLCA [118]. Circ_0068871 increases FGFR3 expression and STAT3 activation by sponging miR‐181a‐5p to promote BLCA [119].

## Role of circRNAs in regulating PDGF signaling in cancer

PDGF exists as a dimer (PDGF‐AA, PDGF‐BB, PDGF‐AB) and interacts with monomeric receptor tyrosine kinases PDGFRα and PDGFRβ to promote receptor dimerization [120]. PDGF signaling activates PI3K/AKT, MAPK/ERK, Notch, and JAK/STAT signaling pathways to promote invasion, angiogenesis, and drug resistance during carcinogenesis [120]. Currently, circRNAs are known to regulate PDGF signaling by modulating PDGF receptor expression (Table 1) [121, 122, 123]. CircCDK14 increases PDGFRA expression by sponging miR‐3938 to promote invasion and reduce sensitivity to ferroptosis in glioma [121]. CircMETRN expression is induced by low‐dose radiation in GBM, increasing PDGFRα levels [122]. It sponges miR‐4709‐3p to alleviate GRB14 expression [122]. GRB14 binds to PDGFRα and positively regulates its expression [122]. In endometrial cancer (EC), circCHD7 enhances the mRNA stability of PDGFRB by interacting with IGF2BP2 [123]. This activates the JAK/STAT signaling pathway via the circCHD7/IGF2BP2/PDGFRB axis to promote EC [123].

## Conclusion

Substantial progress has been made in unraveling the complex interplay of cytokine signaling in cancer [3, 4, 5, 6, 7, 11]. CircRNAs have emerged as an integral part of cytokine signaling cascades. Multiple cytokines such as TGF‐β, IL‐6, and TNF‐α regulate circRNA biogenesis [28, 58, 73, 74, 79, 80, 82]. Aberrantly expressed circRNAs regulate the expression of ligands, receptors, and downstream effectors involved in cytokine signaling (Table 1). Till now, most research on the regulation of cytokine signaling by circRNAs in cancer is focused on tumor‐promoting cytokines (Table 1); it is imperative to understand what role circRNAs play, if any, in regulating the effect of tumor suppressor cytokines such as IFN and IL‐2. Many circRNAs regulate the expression of genes from which they originate [37, 38]. However, it is unclear if circRNAs, apart from circFGFR1 and circFGFR1int2, originate from genes coding ligands and receptors involved in cytokine signaling and, if they do, how they function in cancer [101, 115]. Hence, efforts are still needed to identify and characterize additional circRNAs involved in cytokine signaling. Mechanistically, many circRNAs involved in modulating cytokine signaling act as miRNA sponges, likely due to the relative ease of studying circRNA and miRNA interaction. It is imperative to explore the full spectrum of circRNA mechanisms of action in cytokine signaling, including their roles as protein decoys and scaffolds (Figs 2, 3, 4). Given the multifaceted roles of cytokines in physiology and immune regulation, their complete inhibition may lead to unintended consequences, such as immune suppression or impaired tissue homeostasis [7, 12]. Instead of completely blocking cytokine signaling for cancer therapy, targeting specific circRNAs to inhibit oncogenic activities of cytokine pathways may provide a more refined therapeutic approach. However, the clinical utility of circRNAs as diagnostic and therapeutic targets faces significant challenges due to the lack of standardized detection and validation methods [47, 124, 125]. Furthermore, targeting circRNAs for therapy presents hurdles related to delivery efficiency, specificity, and potential off‐target effects, underscoring the need for further research into optimized RNA‐based therapeutic strategies [124, 125]. Understanding the role of circRNAs in cytokine signaling and advances in therapeutic targeting of circRNAs will likely pave the way for better outcomes for cancer therapy.

## Conflict of interest

The authors declare no conflict of interest.

## Peer review

The peer review history for this article is available at https://www.webofscience.com/api/gateway/wos/peer‐review/10.1002/2211‐5463.70051.

## Author contributions

VJ, S, and VS conceived and designed the manuscript. VJ, S, AM, AP, and VS wrote the manuscript. All authors read and approved the final version of the manuscript.
